# Radiation Damage Mitigation in FeCrAl Alloy at Sub-Recrystallization Temperatures

**DOI:** 10.3390/ma18010124

**Published:** 2024-12-31

**Authors:** Md Hafijur Rahman, Md Abu Jafar Rasel, Christopher M. Smyth, Daudi Waryoba, Aman Haque

**Affiliations:** 1 Department of Mechanical Engineering, The Pennsylvania State University, University Park, PA 16803, USA; mxr5923@psu.edu (M.H.R.); mfr5667@psu.edu (M.A.J.R.); 2 Sandia National Laboratories, Albuquerque, NM 87185, USA; cmsmyt@sandia.gov; 3 Engineering, Applied Materials, The Pennsylvania State University, College Place, DuBois, PA 15801, USA; drw29@psu.edu

**Keywords:** radiation damage mitigation, heavy ion irradiation, FeCrAl alloys, electron wind force, X-ray diffraction, electron backscatter diffraction

## Abstract

Traditional defect recovery methods rely on high-temperature annealing, often exceeding 750 °C for FeCrAl. In this study, we introduce electron wind force (EWF)-assisted annealing as an alternative approach to mitigate irradiation-induced defects at significantly lower temperatures. FeCrAl samples irradiated with 5 MeV Zr^2+^ ions at a dose of 10^14^ cm^−2^ were annealed using EWF at 250 °C for 60 s. We demonstrate a remarkable transformation in the irradiated microstructure, where significant increases in kernel average misorientation (KAM) and low-angle grain boundaries (LAGBs) typically indicate heightened defect density; the use of EWF annealing reversed these effects. X-ray diffraction (XRD) confirmed these findings, showing substantial reductions in full width at half maximum (FWHM) values and a realignment of peak positions toward their original states, indicative of stress and defect recovery. To compare the effectiveness of EWF, we also conducted traditional thermal annealing at 250 °C for 7 h, which proved less effective in defect recovery as evidenced by less pronounced improvements in XRD FWHM values.

## 1. Introduction

The development of advanced nuclear reactors and fusion energy systems necessitates materials that can withstand extreme environments characterized by intense radiation fields and high temperatures [1]. FeCrAl alloys have emerged as strong contenders for fuel cladding application due to their exceptional oxidation resistance and robust mechanical properties at elevated temperatures [2,3]. These alloys are being explored as accident-tolerant cladding materials [4], potentially replacing traditional zirconium-based systems [5]. Additionally, FeCrAl alloys find applications in structural components for fast fission reactors and as first wall and blanket structures for fusion reactors [6]. However, their performance in service environments can be significantly impacted by exposure to radiation. Different types of radiation, such as neutrons, protons, and heavy ions, can introduce defects within the crystal lattice of an alloy [7,8,9,10]. These defects, ranging from vacancies and interstitials to more complex arrangements, can significantly alter the material properties [9,11,12]. Proton irradiation, for instance, can create point defects and displacement cascades, while gamma ray exposure primarily leads to vacancy formation [13]. Heavy ion irradiation also introduces a variety of defects within the lattice, such as vacancies, interstitials, and dislocation loops, which disrupt the orderly atomic arrangement, leading to reduced strength and increased hardness due to dislocation pinning effects [14,15]. Depending on the irradiation dose and environmental conditions, these bombardments can precipitate or form new phases and affect grain size, either refining or coarsening it through interactions with grain boundaries [16,17]. These defects and microstructural changes collectively degrade the alloy’s properties, reducing its creep resistance, which is critical for maintaining structural integrity under high temperature stress and increasing its susceptibility to stress corrosion cracking (SCC) [18,19]. These irradiation-induced challenges necessitate subsequent annealing strategies to restore material properties and extend the service life of structural components.

Thermal annealing has been the traditional method for mitigating defects and damage in metals and alloys. This approach is often contingent on maintaining high temperatures for prolonged periods, with the specific irradiation conditions varying according to the alloy’s composition, as well as the type and dose of radiation received. For instance, solution annealed Inconel 718 requires one hour at temperatures 300–500 °C to reduce for H^+^ or Ni^2+^ irradiation-induced hardening [20]. Further studies affirm that annealing not only diminishes the density of these defects but also influences the behavior and evolution of dislocation loops [21]. Moreover, the efficacy of annealing is influenced by the irradiation conditions, such as the temperature during the irradiation period [22]. High-temperature irradiation has been observed to reduce the internal vacancy defect density due to annealing-induced recovery effects on atomic displacement, particularly in low-Cr FeCrAl alloys, which exhibit a higher vacancy recovery rate compared to higher-Cr content [23]. Annealing efficiency is not a monotonic function of temperature and thus requires careful optimization, particularly in multi-phase alloys where high temperatures can induce undesired phase transformations and grain coarsening [24,25]. For example, in the case of CoCrCuFeNi high entropy alloys (HEAs), thermal annealing has been shown to lead to significant grain coarsening within the nanocrystalline structure, which can adversely affect the material’s mechanical properties and overall structural integrity [26]. Such outcomes highlight the need for alternative annealing strategies that can mitigate radiation damage without the detrimental side effects associated with high-temperature exposure.

EWF is a mechanical stimulus that offers a promising alternative to traditional thermal annealing [27,28,29,30]. This force is generated when current flowing in a material interacts with the defects. The electron loses its momentum to the defect, mobilizing the latter if the energy is more than the activation energy for the defect. No such force is generated due to the electron–lattice interaction, which rather gives rise to Joule heating. Typically, the Joule heating effect overshadows the EWF, known as the electromigration damage phenomenon [31]. However, the EWF can promote migration and annihilation of defects if the Joule heating is suppressed to avoid thermal runaway [31,32]. The most intriguing aspect is the targeted defect recovery with minimal impact on the overall microstructure, since the EWF acts only on the defects and not on the lattice. The primary advantage of EWF is its ability to operate at significantly lower temperatures than conventional methods, reducing the risk of thermal-induced damages such as unwanted phase transformations [33].

The objective of this study was to investigate the effectiveness of EWF annealing in mitigating defects induced by Zr^2+^ irradiation in FeCrAl alloys. Heavy ion irradiation acts as a surrogate for neutron irradiation through displacement damage, which is carefully controlled by the choice of ion beam conditions [34]. One major advantage of heavy ion irradiation is the ability to rapidly accumulate end-of-life doses in short time periods, which is impractical for neutron irradiation. Radiation damage annealing in FeCrAl alloys is not well understood. However, traditional thermal annealing mitigation of radiation-induced defects in FeCrAl alloys generally requires temperatures at or above the recrystallization temperature (≥750 °C) [35].

In this study, we demonstrated mitigation of radiation-induced defects in an FeCrAl alloy at a temperature of 250 °C by implementing the athermal effects of EWF. We conducted comprehensive analyses including electron backscatter diffraction (EBSD) with a focus on KAM maps and XRD to evaluate the effectiveness of EWF treatments at eliminating radiation-induced damage. We hypothesize that EWF mitigates radiation-induced defects by enhancing the mobility of defects with minimal thermal contribution, which may serve as a more energy-efficient and less invasive recovery process.

## 2. Materials and Methods

**Figure 1:**
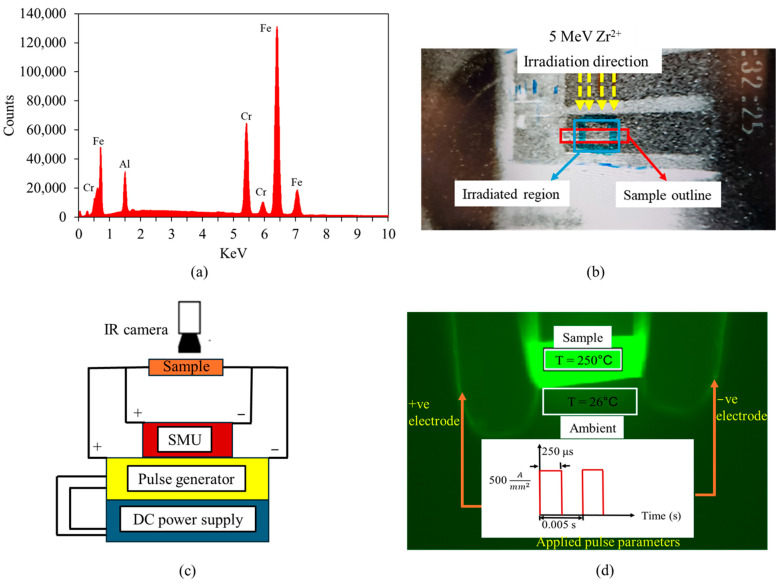
(**a**) Energy dispersive X-ray spectrum confirming alloy elements in the specimen, (**b**) heavy-ion irradiation condition, (**c**) schematic diagram of EWF annealing setup, (**d**) thermal image of the sample along with associated processing parameters.

In this study, an FeCrAl alloy was irradiated with 5 MeV Zr^2+^ ions with dose of 10^14^ cm^−2^. and subsequently subjected to a series of EWF treatments to assess its effectiveness towards athermal defect annealing. The composition of the FeCrAl alloy (Fe-73%, Cr-21.3%, Al-5.7%) was measured using energy dispersive X-ray (EDX) analysis (see Figure 1). FeCrAl alloys with a similar elemental ratio have been extensively studied and used in nuclear systems due to their exceptional oxidation resistance and mechanical properties [36]. The 5 MeV Zr^2+^ ion irradiation was conducted at the Ion Beam Laboratory at Sandia National Laboratories using a 6 MV tandem accelerator. Following irradiation, the samples were subjected to EWF annealing facilitated by a programmable DC power supply (Magna-power, SL600-2.5/UI, Flemington, NJ, USA) and a current pulse generator (IPM-16P-2003, Eagle Harbor Technology, Seattle, WA, USA). Specimen temperature during the process was continuously monitored using a thermal microscope (Optris PI 640, Portsmouth, NH, USA). Initially, the EWF annealing parameters (current, pulse width, frequency) were selected to utilize the potential of the EWF while minimizing the thermal effects. We began with a low frequency and pulse width of 2 Hz and 40 µs, respectively, to emphasize the athermal effects of the EWF. To monitor the microstructural changes, the current density was incrementally increased, with continuous resistance measurements conducted using a Keithley 4200 (Solon, OH, USA) SMU. At a current density of 500 A/mm^2^, minor changes in resistance suggested the onset of microstructural modifications. However, drawing from insights in our previous study [33], which demonstrated effective defect mitigation by combining EWF and thermal effects, we opted not to further increase the current density. Instead, we increased both the frequency (200 Hz) and pulse width (250 μs) to induce a controlled rise in temperature. This adjustment led to a significant resistance change at 250 °C, indicating pronounced microstructural changes. The annealing time was kept fixed to 60 s for all cases since most of the annealing effect takes place in the first few pulses.

Post-annealing microstructural evaluations were conducted using XRD and electron backscattered diffraction (EBSD) analyses. It is expected that ion irradiation would produce extensive point defect clusters to induce swelling in the material (captured with XRD) with some increase in low angle grain boundaries originating from dislocation loop entanglement (captured with EBSD). The XRD characterization was carried out with a Malvern Panalytical Empyrean^®^ (Westborough, MA, USA) diffractometer, equipped with a cobalt line-focus X-ray tube (wavelength = 1.7889 Å), and operated at optimal settings (40 kV and 40 mA) for comprehensive sample irradiation. The configuration included a divergence slit of 1/16°, a 2 mm mask, a soler slit of 0.04 rad, and an anti-scatter slit of 1/4°. The 2θ scan range spanned from 35° to 125° with a step size of 0.016°, which is optimized to maximize the detection sensitivity for subtle shifts in peak broadening indicative of residual stress and defect transformations resulting from the ion irradiation and EWF treatments. We also performed micro-XRD using a Cu source on the thermally annealed and EWF annealed samples to compare the annealing efficacy. The detailed parameters for micro-XRD are given in Section 4. All post-analysis, including the determination of peak positions and full width at half maximum (FWHM) values, was performed using Jade^®^ software (version 8.9) from Materials Data Inc. (Livermore, CA, USA). It is to be noted that we observed multiple reflections within each peak, even for the pristine sample. This can be attributed to intrinsic residual stresses present within the alloy, likely introduced during the fabrication process. Further details regarding the observed reflections and the methodology employed for peak selection and FWHM determination are provided in the Supplementary File. EBSD, on other hand, directly visualizes the microstructure and allows mapping of low angle grain boundaries and internal stresses to assess the impact of irradiation and subsequent annealing on defect structures. Prior to EBSD analysis, extensive surface preparation was undertaken, involving mechanical polishing through a sequence of diamond compounds (from 3000 to 120,000 grit) followed by ion milling at 4.5 kV and 1.5 A for 50 min. The EBSD scans were performed using a VERIOS G4 UC Scanning Electron Microscope (Thermo Scientific, Hillsboro, OR, USA), under a beam current of 3.2 nA and an accelerating voltage of 20 kV, with a step size of 0.3 µm. The obtained data were processed using the Aztec software suite (version 6.2 for acquisition, version 5.1 for post-processing) [37].

## 3. Results

Exposure to high-energy radiation such as 5 MeV Zr^2+^ ions with dose of 10^14^ cm^−2^ introduces a variety of microstructural defects, including vacancies, interstitials, and complex dislocation networks, which can degrade mechanical properties and enhance susceptibility to damage. Effective mitigation of radiation-induced defects is essential for maintaining performance and extending service life in harsh environments. In this section, we explore the transformative effects of the EWF athermal annealing on FeCrAl alloys that have undergone 5 MeV Zr^2+^ irradiation. 

### 3.1. Stopping and Range of Ions in Matter Simulations

**Figure 2:**
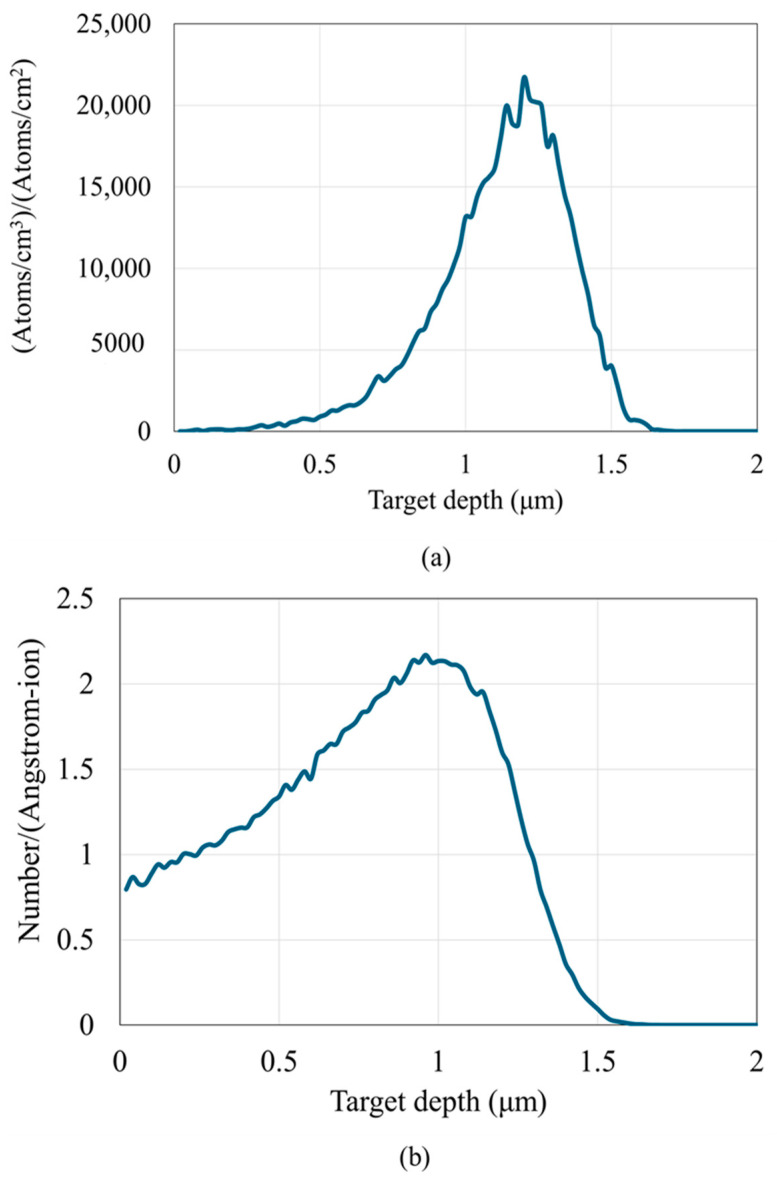
(**a**) Ion distribution and (**b**) collision event (vacancy produced using the Kinchin–Pease model) for Zr^2+^ irradiated at 5 MeV with dose of 10^14^ cm^−2^ in FeCrAl alloy.

We employed stopping and range of ions in matter (SRIM) simulations to understand the impact and distribution of defects induced by 5 MeV Zr^2+^ with a dose of 10^14^ cm^−2^ in FeCrAl alloys. As illustrated in Figure 2, the ion distribution and collision events (effective displacement per atom is reported) show that the penetration depth of Zr^2+^ ions is less than 2 µm (Figure 2a), and the maximum damage occurs at approximately 1.11 µm (Figure 2b). At a depth of ~1 µm, the vacancy production rate peaks at 2.1 vacancies per target atom. Stable displacement damage resulting from radiation at room temperature has been reported to constitute only a small fraction of the initially produced damage [38]. Based on this, we have assumed that most of the defects recombine or anneal immediately, leaving only 1% as stable damage in the lattice, leading to a vacancy density of approximately 2 × 10^20^ vacancies per cubic centimeter.

### 3.2. X-Ray Diffraction (XRD) Analysis

**Figure 3:**
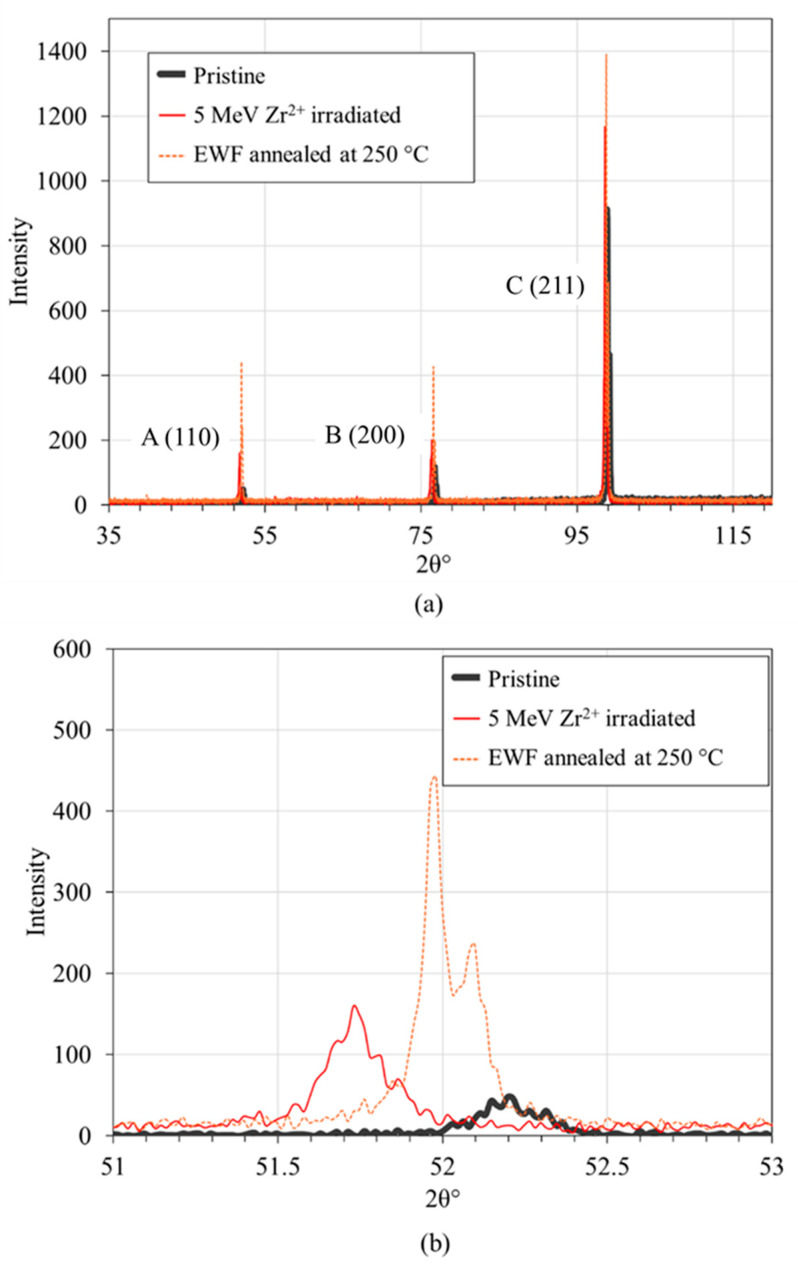

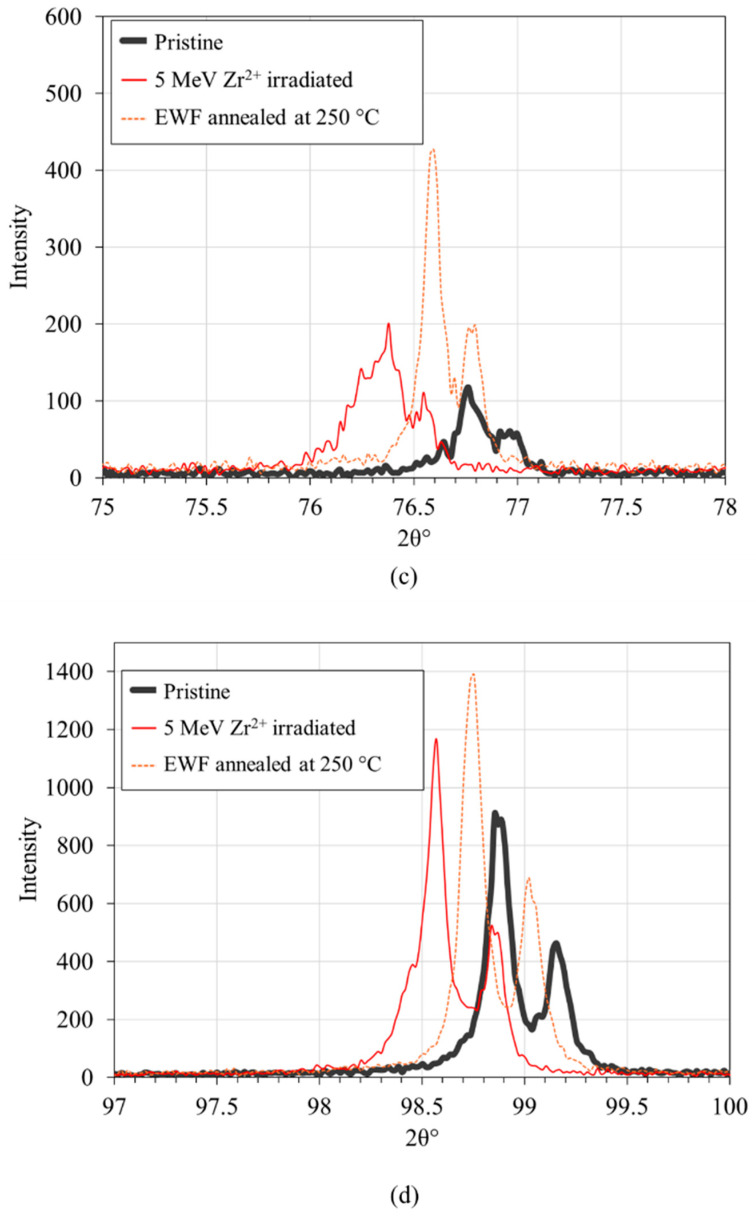
XRD patterns of FeCrAl alloy across different conditions: (**a**) complete spectrum from 35° to 125°, highlighting three major peaks identified as peak A, peak B, and peak C; (**b**), (**c**), and (**d**) detailed views of peak A, peak B, and peak C, respectively.

XRD was employed to investigate the effect of EWF annealing on the irradiation damage inflicted by the Zr^2+^ ions. The pristine FeCrAl sample exhibited three distinct diffraction peaks labeled A, B, and C represent the (110), (200) and (211) planes, respectively (Figure 3). These three peaks are characteristic of the alloy’s body-centered cubic (BCC) structure. The position of three peaks for various conditions are shown in Table 1. Upon Zr^2+^ irradiation, the FWHM of these peaks underwent noticeable changes, with the peak A FWHM increasing from 0.189° to 0.21°, peak C FWHM increasing from 0.133° to 0.16°, and a more substantial increase in the peak B FWHM from 0.22° to 0.55°. Table 2 shows the changes in FWHM of peaks A, B, and C upon irradiation and EWF treatment is shown in Table 2. The measured FWHM increase is indicative of increased microstructural disorder and defect density, primarily attributed to increased dislocation density and the introduction of point defects, such as vacancies and interstitials [39,40]. Concurrently, a leftward shift of all three peaks was detected after radiation. Peak shift to the left indicates tensile stress in the specimen originated from swelling induced lattice expansion [6,41,42] due to the ion irradiation. It is likely that the combination of vacancies and dislocations synergistically contributes to the observed peak broadening and shifting. Dislocations, as linear defects, introduce significant lattice distortions, leading to increased peak broadening. Vacancies, while individually having a smaller impact, can contribute to overall lattice strain when present in high concentrations, as in the case of heavy ion irradiation.

After EWF-assisted annealing, the FWHM values for peaks A, B, and C were reduced to 0.128°, 0.135°, and 0.133°, respectively, suggesting significant defect recovery and microstructural recovery near to the original state. Moreover, all diffraction peaks detected by XRD exhibit a positive shift nearly to their initial positions. The upward shift following annealing suggests lattice contraction, possibly associated with the annihilation of induced defects. These XRD findings suggest that Zr^2+^ irradiation induced significant microstructural alterations, including lattice strain and potential defect accumulation. The subsequent EWF annealing process effectively mitigated these irradiation-induced changes, restoring a microstructure closer to the pristine state.

### 3.3. Electron Backscatter Diffraction Characterization

#### 3.3.1. Kernel Average Misorientation (KAM) Maps

**Figure 4:**
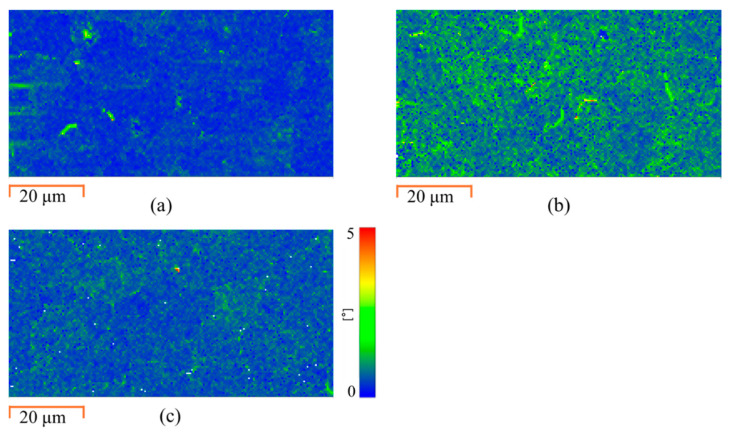
Kernel average misorientation maps for (**a**) pristine, (**b**) irradiated, (**c**) EWF annealed conditions.

We utilized EBSD to characterize grain size, texture and dislocation density changes in the FeCrAl alloy post-irradiation with Zr^2+^ ions and subsequent annealing using the EWF at 250 °C. Radiation-induced defects are often detected as changes in local crystallographic misorientation [19,43,44]. The KAM values, derived from the EBSD data, provide a quantitative measure of these local misorientations, which are directly related to the geometrically necessary dislocations within the material [43,45]. For pristine specimens, the dislocation density is minimal, as shown in Figure 4a.

After irradiation, KAM analysis shows a significantly increased density of local dislocation networks (Figure 4b), which confirms the substantial defect density introduced by the ion irradiation [46,47]. After athermal EWF annealing at 250 °C, the KAM analysis reveals a marked decrease in dislocation density (Figure 4c). This decrease indicates that the EWF-assisted annealing eliminated most radiation-induced dislocations. This result highlights the effectiveness of EWF in mitigating irradiation-induced defects well below the alloy recrystallization temperature and identifies a low-temperature alternative to traditional high-temperature annealing.

#### 3.3.2. Low-Angle Grain Boundaries (LAGBs)

**Figure 5:**
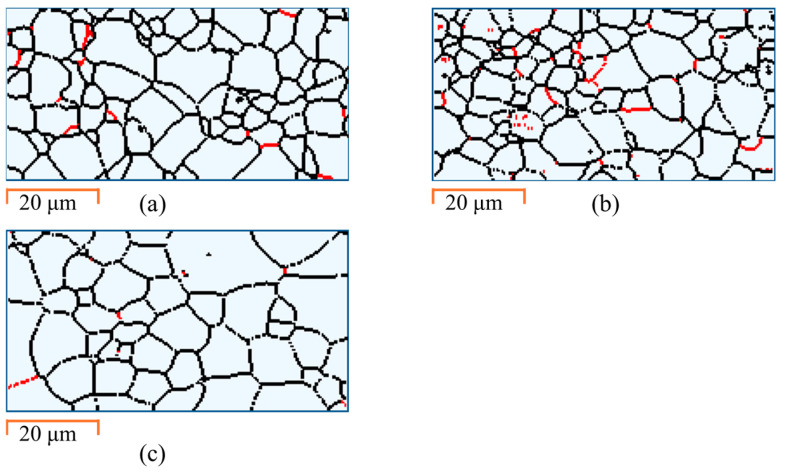
Grain boundaries for (**a**) pristine, (**b**) irradiated, (**c**) EWF annealed at 250 °C. Red color indicates low angle grain boundaries-LAGBs (2° ≤ θ ≤ 10°) and black color indicates high angle grain boundaries (θ > 10°).

Following heavy ion irradiation, the FeCrAl alloy exhibited a significant transformation in its microstructural characteristics, particularly in the form of LAGBs. Prior to irradiation, a LAGB concentration of 4.5% is measured (see Figure 5a). Radiation-induced defects are typically accommodated within grains as LAGBs [48,49]. These LAGBs form due to the accumulation and interactions of dislocations, creating boundaries with small misorientations (<10°) between adjacent crystalline areas. Following the Zr^2+^ irradiation, a significant increase in the concentration of LAGBs to 11% is measured (see Figure 5b). The increased LAGB concentration measured after irradiation agrees with the compressive strain and increased disorder measured by XRD.

The application of EWF annealing at 250 °C effectively addressed these radiation-induced defects. After undergoing EWF-assisted annealing, the concentration of LAGBs was significantly reduced to 1.7% (see Figure 5c), which is even lower than that in the pristine condition. This notable decrease in LAGB concentration suggests that the EWF annealing not only relieved the irradiation-induced stress and dislocation density but also improved the overall microstructural stability of the alloy, surpassing its initial undamaged state. By reducing the LAGB concentration below the initial baseline, EWF annealing demonstrated its capability to refine the grain structure and diminish residual stresses, contributing to the improved mechanical integrity and performance of the alloy. Similar results have been reported in our previous studies on mitigating cold-rolling induced defects [27,33].

## 4. Comparative Analysis of EWF and Thermal Annealing

**Figure 6:**
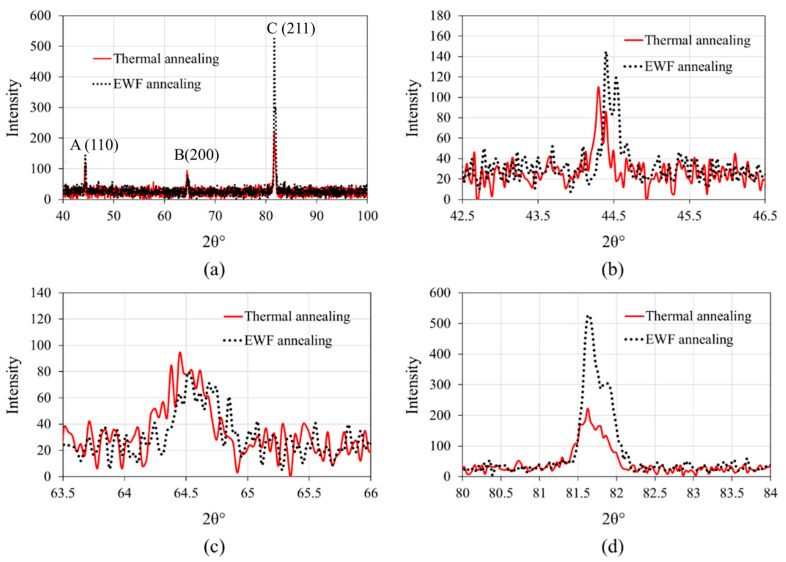
(**a**) XRD analysis of thermally annealed and EWF annealed samples; (**b**), (**c**) and (**d**) are the zoomed-in view of peaks A, B and C, respectively.

We conducted a comparative analysis to evaluate the effectiveness of EWF annealing against traditional thermal annealing in mitigating defects in FeCrAl alloys irradiated with 5 MeV Zr^2+^ ions. Both sets of samples were annealed at 250 °C; however, for the thermal annealing, the samples underwent a more prolonged treatment—7 h total, involving a ramping up at 10 °C per minute to 250 °C, holding at this temperature for 1 h, and then a gradual cooldown for the remainder. In contrast, EWF annealing was completed within just 1–2 min, demonstrating a substantial reduction in processing time. Subsequently, XRD analysis was performed (Figure 6) to assess the changes in microstructure and defect states between the two annealing methods. For this part, XRD was performed using a point focus on a Panalytical Empyrean^®^ theta-theta X-ray diffractometer (Malvern Panalytical Ltd, Malvern, UK). Operating conditions were set at 40 kV and 40 mA, utilizing a Cu K-α X-ray tube with wavelengths of 1.540598 Å and 1.544426 Å. The data collection spans a 2θ range from 40° to 100° with a fine step size of 0.033° to precisely capture peak shifts and broadenings indicative of microstructural changes. The sample was accurately positioned using a programmable x-y-z stage, with the incident beam focused on a 0.3 mm mono-capillary optic. The diffracted beam was processed using an X’Celerator^®^ detector, employing a 1/4° programmable anti-scatter slit and 0.04 rad Soller slits, paired with a nickel K-beta filter to enhance measurement precision and reduce noise.

The XRD results reveal distinct differences in the effectiveness of defect mitigation between EWF annealing and thermal annealing, as evidenced by the FWHM values for the three main peaks. It is to be noted that there are small variations in diffraction peak positions between Figure 3 and Figure 6, which arise due to the use of two distinct XRD setups: a cobalt source for the primary measurements in Figure 3 and a copper source for micro-XRD analysis (Figure 6) conducted to accommodate sample size constraints during thermal annealing experiments. While both methods achieved comparable recovery for Peak A (FWHM = 0.077°), corresponding to the (110) plane, significant differences were observed for Peaks B and C, representing the (200) and (211) planes, respectively. For the thermally annealed samples, the FWHM values for Peak B and Peak C were 0.313° and 0.273°, respectively, reflecting a higher residual defect concentration and strain in the crystal lattice. In contrast, the EWF annealed samples demonstrated substantially lower FWHM values of 0.218° for Peak B and 0.198° for Peak C. These results suggest that while thermal annealing effectively addresses point defects and minor lattice distortions in the (110) plane, it falls short in mitigating more complex defects, such as dislocation loops or defect clusters, which predominantly affect the higher-index (200) and (211) planes. The (200) and (211) planes are less densely packed compared to the (110) plane, making them more susceptible to complex defects like dislocations or clusters of vacancies. These defects are more difficult to recover fully during thermal annealing, especially if the annealing temperature is not sufficient to remove them. In this case, the thermal annealing at 250 °C was insufficient for comprehensive defect recovery, primarily because this temperature is well below the 750 °C recrystallization threshold of FeCrAl [50]. At such a low temperature, the diffusion processes necessary for defect mitigation are not fully activated. This leads to inadequate defect recovery, as the atomic mobility required to facilitate defect recombination and annihilation is significantly reduced. On the other hand, despite the same peak temperatures, EWF annealing was more effective in reducing these complex defects than thermal annealing, as evidenced by better recovery in FWHM values of peaks B and C. As we discussed earlier, EWF annealing incorporates not only the thermal effects due to joule heating but also the athermal effects from the EWF. This combination enhances atomic mobility and defect interactions even at lower temperatures, thereby effectively reducing the dislocation density and mitigating radiation-induced damage. This synergistic approach not only significantly reduces processing time but also surpasses the effectiveness of conventional annealing, offering a compelling alternative for enhancing the material properties of irradiated FeCrAl alloys.

## 5. Conclusions

In this study, we investigated the effect of EWF assisted annealing on FeCrAl alloys that had been subjected to 5 MeV Zr^2+^ irradiation. The primary aim was to evaluate whether EWF annealing can be more effective in irradiation damage mitigation compared to traditional high temperature annealing. We present evidence of enhanced recovery with EWF annealing at 250 °C and 60 s, effectively mitigating the defects induced by irradiation. After irradiation, the microstructural changes were significant: there was an increase in KAM, indicating enhanced dislocation activity; the LAGBs concentration rose, reflecting the enhanced accumulation of dislocations; FWHM values across key diffraction peaks increased, suggesting increased defect densities; and there was a notable leftward shift in peak positions in the XRD profiles, indicative of the stress and defects induced by irradiation. Our results demonstrate that EWF-assisted annealing significantly reduced the defect density in the irradiated FeCrAl samples. We observed a marked decrease in LAGBs and KAM values post-annealing, indicating effective defect recovery. Specifically, the concentration of LAGBs was reduced to levels even lower than those in the pristine state, highlighting the potency of EWF in refining the microstructure. XRD analysis further substantiated these findings, showing a normalization of peak broadening and a reversion of peak shifts toward their original positions after annealing, suggesting a reversion toward the original lattice parameters and a decrease in residual stress and strain within the alloy. On the other hand, thermal annealing at 250 °C failed to revert the peak broadening and shifts to their original states, illustrating its inadequacy at this lower temperature, which is far below the alloy’s recrystallization threshold of 700 °C.

## Figures and Tables

**Table 1:** Position in degree (°) unit of three peaks detected by XRD throughout the experiment.

	Peak A	Peak B	Peak C
Pristine	52.194	76.764	98.861
5 MeV Zr^2+^ irradiated	51.729	76.37	98.569
EWF annealed at 250 °C	51.99	76.6	98.76

**Table 2:** Full width at half max (FWHM) in degree (°) unit of three peaks for various conditions.

	Peak A	Peak B	Peak C
Pristine	0.189	0.22	0.133
5 MeV Zr^2+^ irradiated	0.21	0.515	0.166
EWF annealed at 250 °C	0.128	0.135	0.133

## Data Availability

The original contributions presented in this study are included in the article/Supplementary Material. Further inquiries can be directed to the corresponding author.

## Supplementary Materials

The following supporting information can be downloaded at: https://www.mdpi.com/article/10.3390/ma18010124/s1, Figure S1: Methods used to determine peak position and FWHM values of a peak using Jade^®^ software (version 8.9).
