# Supplementary material for: Radiation Damage Mitigation in FeCrAl Alloy at Sub-Recrystallization Temperatures
Source: Materials (Basel). 2024 Dec 31;18(1):124. doi: 10.3390/ma18010124 (PMC11721584; doi:10.3390/ma18010124)
Supplement: Supplementary file 1 [file materials-18-00124-s001.zip › materials-3352069-supplementary.pdf]

The Jade® software used for data analysis designates the reflections as separate peaks. To maintain consistency in the analysis, we identified the reflection with the maximum intensity as the representative peak. The reported peak position and Full Width at Half Maximum (FWHM) values correspond to this representative peak. For example, in Figure 3d, the peak position and FWHM values of peak C are determined as followings:

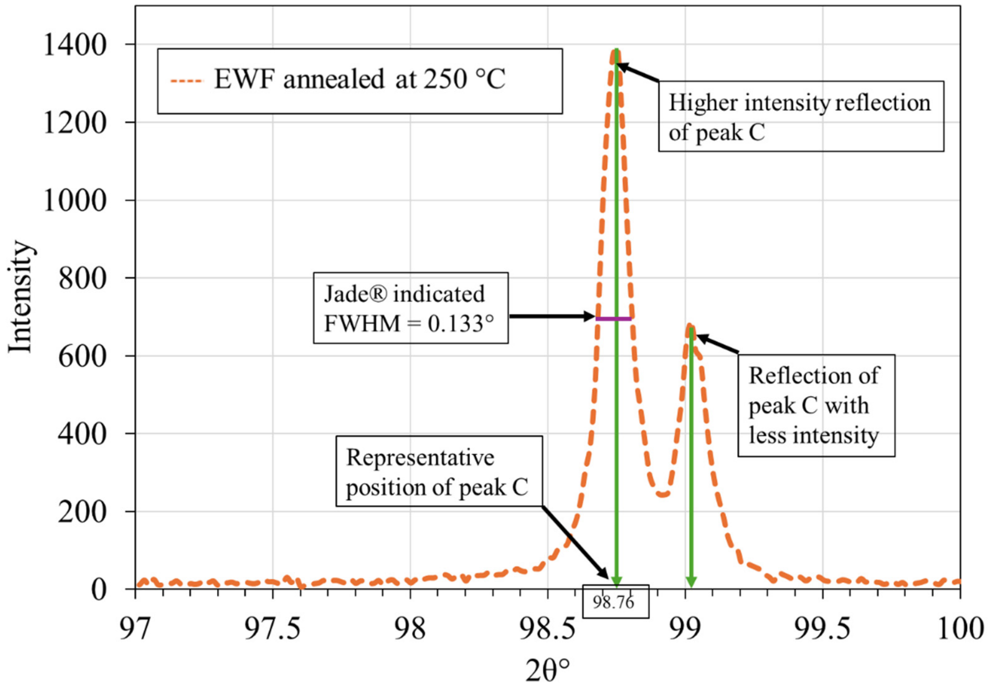

Supplementary Figure S1: Methods used to determine peak position and FWHM values of a peak using Jade® software (version 8.9).
